# Blockchain technology in the pharmaceutical industry: a systematic review

**DOI:** 10.7717/peerj-cs.840

**Published:** 2022-03-11

**Authors:** Nazik Zakari, Muna Al-Razgan, Amani Alsaadi, Haya Alshareef, Heba Al saigh, Lamia Alashaikh, Mala Alharbi, Rana Alomar, Seham Alotaibi

**Affiliations:** 1 Nursing Department, College of Applied Sciences, Al Maarefa University, Riyadh, Saudi Arabia; 2 Department of Software Engineering, College of Computer and Information Sciences, King Saud University, Riyadh, Saudi Arabia

**Keywords:** Blockchain, Pharmaceutical industry, Drug traceability, Counterfeit drugs, Drug safety and security, Systematic literature review

## Abstract

Blockchain technology is accelerating digital transformation across multiple industries, including the pharmaceutical industry. The pharmaceutical industry suffers from a lack of transparency, difficulty tracking products, lack of trust, and the shipment of expired products. Blockchain technology has been applied to solve several of these problems. In this paper, we present a systematic review of the literature focusing on the adoption of blockchain technology in the pharmaceutical industry. We collected, analyzed, qualified, and discussed studies retrieved from seven databases. The initial search yielded 2,185 papers, which were screened, discussed, voted on, critically appraised, and collected by a snowball workflow that finally yielded 38 papers. The blockchain application areas covered in the papers were classified as counterfeit drug prevention, drug distribution, tracking and tracing, and safety and security. The most frequent category was counterfeit drug prevention, which is consistent with the primary objective of the pharmaceutical industry. The newer topics discussed in this study were data governance, data quality, pharmaceutical turnover, and prescription drug monitoring. We discuss issues surrounding each of these topics and research studies, along with their limitations and solutions. We also examine the challenges and future research directions of applying blockchain technology in the pharmaceutical industry.

## Introduction

In recent years, the proliferation of research, projects, and discussions of blockchain technology has attracted the attention of both researchers and practitioners. Blockchain technology is a distributed ledger that enables the efficient, permanent, and verifiable recording of transactions. It is also a decentralized database that can be used to manage an ever-growing list of records called nodes (Soundarya, Pandey & Dhanalakshmi 2018). One of the advantages of blockchain technology is the inability to change any contracts and transactions that are added to the ledger. Blockchain technology can be used in any industry that requires procedures to be verified and recorded, as well as industries in which sensitive data are managed, including healthcare, medical research, military, banking, finance, and insurance (Bera et al., 2020; Bhardwaj et al., 2020; Kumar et al., 2021).

**Figure 1:**
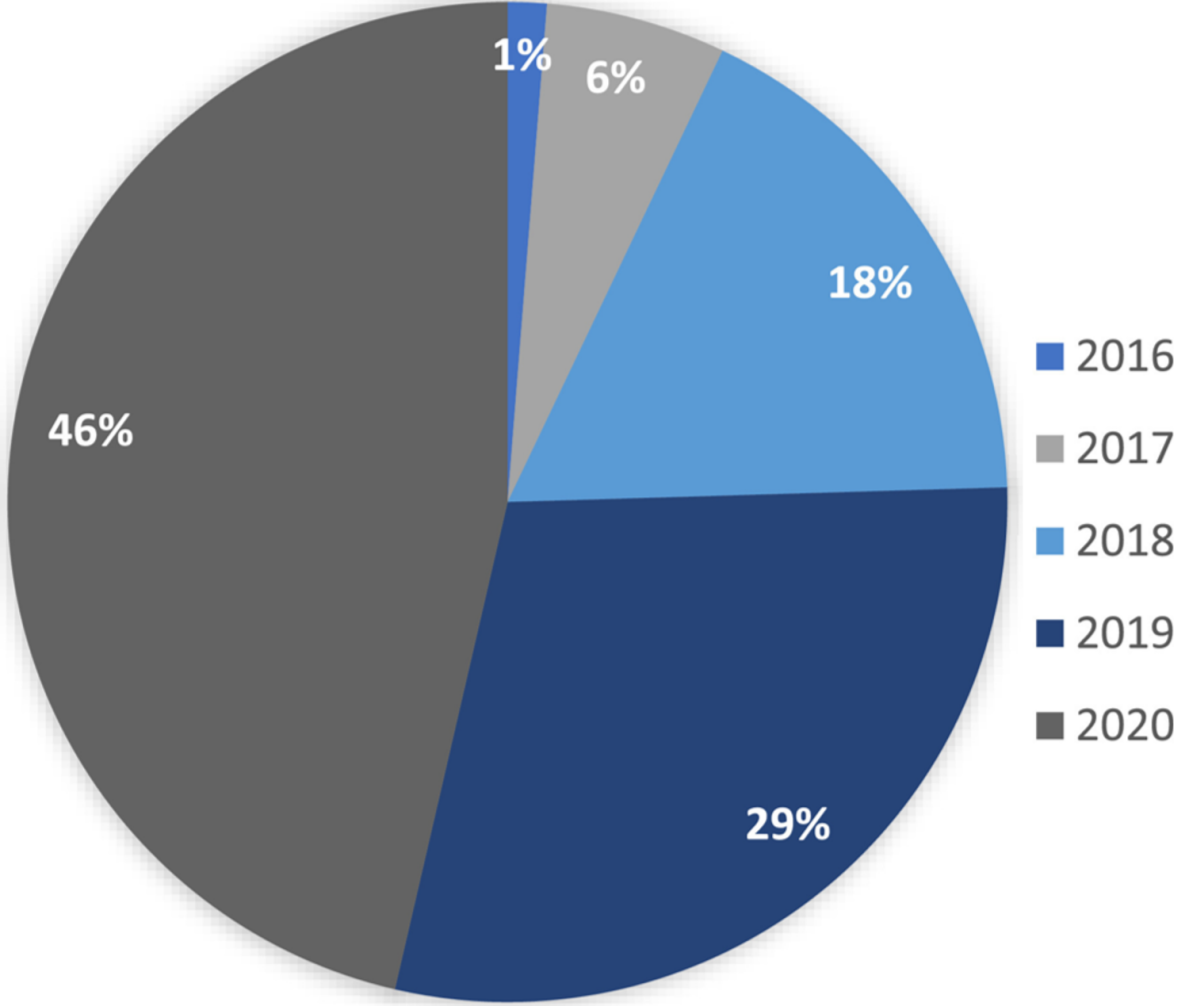
Proportions of research on blockchain in the healthcare sector.

**Figure 2:**
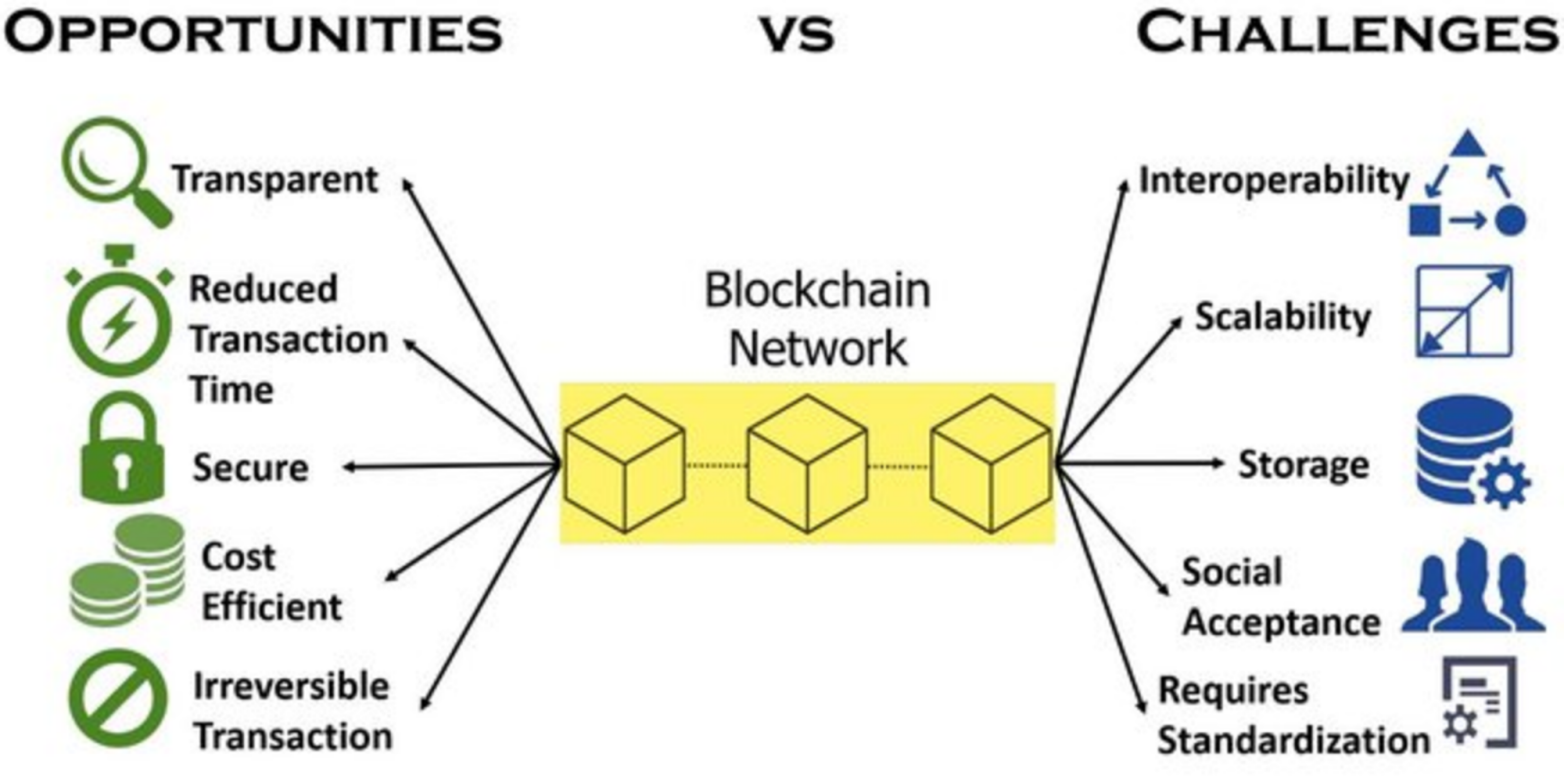
Advantages and challenges related to the application of blockchain.

Figure 1 shows the percentage distribution of research articles on blockchain that have been published in the healthcare industry. As the figure indicates, the number of articles published in this area has increased rapidly year on year. Also, Fig. 2 shows the advantages associated with applying blockchain technologies, as well as the related challenges.

The traditional pharmaceutical supply chain faces a range of challenges, including lack of transparency, difficulty tracking products, lack of trust, and the shipment of expired products (Sinclair, Shahriar & Zhang, 2019). Nevertheless, the pharmaceutical industry must maintain reliable information about the origins of raw materials through drug production and distribution (Plotnikov & Kuznetsova, 2018; Huang, Wu & Long, 2018; Haq & Muselemu, 2018; Garankina et al., 2018).

Counterfeit drugs are produced outside the legitimate pharmaceutical manufacturing system and, therefore, are fraudulent. Given that counterfeit drugs resemble the original drugs, they can be challenging to detect. In most cases, they are designed to appear identical to the original product and may not cause an obvious or harmful reaction. However, they often fail to treat the disease or ailment for which they were designed in an effective way, or they may contain harmful and sometimes toxic ingredients. Pharmaceutical drugs are also used in the diagnosis, treatment, and prevention of disease. In recent years, counterfeit drug production has been a growing industry, and counterfeit drugs result in an estimated 100,000 to 1,000,000 deaths annually (Jamil et al., 2019; Anonymous, 2018). Counterfeit drugs affect the reputations and revenues of legitimate drug manufacturers, but the prevention of counterfeits from entering the market is also urgently needed to safeguard patients.

Given the properties of blockchain technology, particularly the way it enables decentralization, transparency, trust, anonymity, and stability, the use of blockchain technology in the pharmaceutical industry has been identified as a viable way to protect against counterfeit drug distribution. Blockchains can be used to trace the origin of pharmaceuticals, the transport of drugs, and the procurement of raw materials. Blockchain technology also reduces the number of intermediaries involved in the pharmaceutical process, thereby reducing costs and improving safety (Huang, Wu & Long, 2018).

In this paper, we present a systematic literature review (SLR) focusing on the use of blockchain technology in the pharmaceutical industry, including in applications such as tracking and tracing, counterfeit prevention, distribution, and data security. The review includes 38 articles published between 2016 and 2021. The most recent literature review of blockchain technology applications in the pharmaceutical industry was a meta-analysis of 15 articles, which identified factors influencing the successful use of the technology (Fernando, 2019; Fernando et al., 2018). However, the study was limited and did not provide an in-depth analysis of the limitations and challenges of blockchain adoption, nor did it discuss future research directions.

Therefore, this SLR provides an overview of recent research to facilitate an understanding of blockchain technology applications in the pharmaceutical industry, as well as the future of blockchains in this area. Given the unique benefits of blockchains in promoting traceability, transparency, and trust among pharmaceutical stakeholders, it is essential for academics, practitioners, and the healthcare industry as a whole to understand the technology.

In this SLR, we also examine the blockchain frameworks that have been developed in the pharmaceutical industry. In particular, we discuss white papers and vision papers that have covered the use of blockchain in the pharmaceutical industry. We also identify the limitations associated with blockchain research in the pharmaceutical industry. The challenges and solutions of blockchains applications in complex environments (*e.g.*, big data) are also discussed.

This SLR of blockchain technology in the pharmaceutical industry addresses the following questions:

• RQ1: What blockchain technologies are used in the pharmaceutical industry?
• RQ2: Which parts of the industry has blockchain been applied to?
• RQ3: What are the current research limitations?
• RQ4: What are the future research directions?

This study has the following highlights:

1. A systematic literature review of blockchain technology in the pharmaceutical industry, including 38 articles published between 2016 and 2021.
2. In-depth analysis of the limitations and challenges of blockchain adoption, along with a discussion of these and an assessment of future directions.
3. Analysis of white papers and vision papers covering the use of blockchain technology in the pharmaceutical industry.
4. Identification of the limitations associated with blockchain research in the pharmaceutical industry.
5. Discussion of the challenges and solutions of blockchain applications with big data.

This paper is organized as follows: ‘Related Work’ summarizes related work; ‘Blockchain Technology’ describes the basics of blockchain technology; ‘Review Methodology’ presents the SLR’s methodology; ‘Results’ describes the SLR’s results; ‘Discussion’ discusses the results; and ‘Conclusions’ presents concluding remarks.

## Related Work

Only one previous literature review has been undertaken that focuses on blockchain technology applications in the pharmaceutical industry (Fernando, 2019). With the exception of Fernando (2019), no other work based on a literature review was identified in this area, and no studies were found that addressed the issues, challenges, and solutions related to the application of blockchains in the pharmaceutical industry. Hence, our work is the first comprehensive literature review in this area that covers important blockchain technologies in the pharmaceutical industry, different crucial areas of the pharmaceutical industry, and current research limitations, challenges, solutions, and future directions.

The literature review presented in Fernando (2019) sought to identify the success factors for applying blockchain technology in the pharmaceutical industry. The authors used a meta-analysis process for 15 articles and reported on 21 success factors, out of which they selected the five most important: namely, trust, tracking, transparency, traceability, and real-time. Notably, these five factors were discussed superficially without any mention of the applications of blockchain or the issues related to the domain.

Another review article (Alshahrani & Alshahrani, 2021) discussed the assessment of blockchain applications to improve the pharmaceutical industry. The authors used quantitative analysis methods to collect data. They found that the main hindrances to blockchain application in the pharmaceutical industry in Saudi Arabia were healthcare professionals’ perceptions, lack of cooperation, and economic inequality. The authors also identified factors that could facilitate blockchain applications, including system robustness, data safety, improved supply chain management, decentralization, interoperability, and government policies and laws. Significantly, similar to Fernando (2019), Alshahrani & Alshahrani (2021) did not discuss anything related to blockchain technology, issues, and challenges.

In Uddin et al. (2021), the authors discussed the architectures and challenges of using blockchain for drug traceability. They discussed issues related to product traceability in the pharmaceutical supply chain and highlighted solutions for the effective use of blockchain technology in tracking and tracing to mitigate counterfeit medications. Apart from the pharmaceutical supply chain, the authors did not discuss blockchain technology or the issues and challenges related to other domains of the technology’s applications in the pharmaceutical industry.

**Figure 3:**
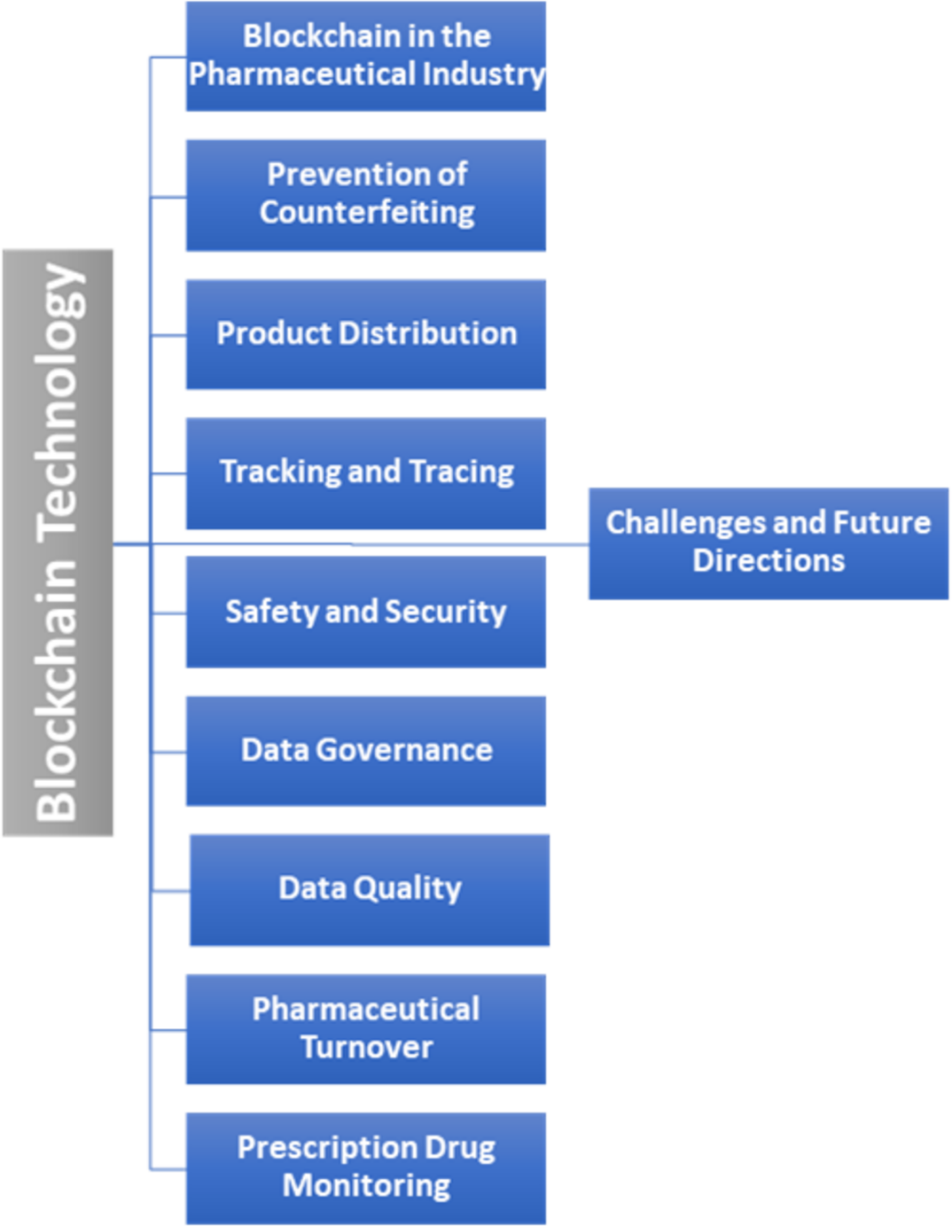
Taxonomy of the literature review.

Importantly, all of the above-mentioned studies offered only a limited and superficial overview of blockchain applications, which highlights the need for a comprehensive literature review in this area. Therefore, our SLR accounts for this gap by providing an in-depth analysis of the limitations and challenges of blockchain technology adoption in the pharmaceutical industry, as well as associated challenges, solutions, and future directions. The taxonomy of this literature review is presented in Fig. 3.

## Blockchain Technology

A blockchain is a tamper-proof, distributed database that stores blocks of information for cryptographically bound transactions *via* peer-to-peer networks. The architecture of a blockchain enables users to share validated and updated ledgers for every transaction. Nodes receive copies of each transaction and validate them, and this information is synchronized throughout the network. As such, the need for a central validating authority is eliminated. The connections between transactions in separate blocks are linked, which is what gave rise to the term “blockchain” (Soundarya, Pandey & Dhanalakshmi 2018).

An essential focus of the pharmaceutical industry is to ensure rapid and secure transactions, along with supply chain fidelity. Stakeholders in the pharmaceutical industry frequently suffer losses *via* theft and loss of goods due to a lack of robust tracking and tracing (Haq & Muselemu, 2018). Drug and medical regulatory authorities have also discovered substandard and counterfeit products in the marketplace. Therefore, many organizations in the pharmaceutical industry, as a result of malpractice and poorly functioning supply chains, have sought to employ blockchain technology to harness operations and streamline tracing and tracking, medical transactions, and patient safety (Huang, Wu & Long, 2018).

### Blockchain technology in the pharmaceutical industry

Blockchains are used in the pharmaceutical industry for different purposes. Security, which is a major challenge, is addressed through the use of cryptographic technologies that validate blocks of transactional data (Sinclair, Shahriar & Zhang, 2019). Security elements unfold in different paradigms. Falsified medications are a security threat that has been addressed through serialization (Alshahrani & Alshahrani, 2021). In this technology, the entire supply chain system is equipped with verification checks that authenticate serial numbers. Drug traceability has also been enhanced to prevent theft (Huang, Wu & Long, 2018), and quality control is achieved through the use of digital signatures; blockchain chaincodes, health information, and data miners are deployed from the manufacturer to the pharmacy to maintain consistent quality (Makarov & Pisarenko, 2019).

### Counterfeit prevention

Pharmaceutical products are serialized and assigned security features that can be verified by consumers and differentiated from counterfeits. The blockchain system also enhances security through transparent and chaincode-based transactions. Trust and transparency are necessary for the pharmaceutical industry because, without trust, the counterfeiting business thrives, exposing the public to dangers arising from low-quality or substandard pharmaceuticals. When blockchains are included in quality control and the detection of counterfeit drugs, this enhances safety and saves lives (Adsul et al., 2020). Several approaches, including the Anti-Counterfeit Medicine System (ACMS), can be used to prevent counterfeiting. ACMS uses the Interplanetary File System (IPFS) networks and the Ethereum blockchain as follows:

• Establish ownership criteria for retail and non-retail medicines to prevent cloning
• Build Ethereum smart contracts for practical ACMS management, exploiting IPFS networks and Ethereum blockchain
• Implement the program for small firms
• Evaluate and analyze the proposed system (Saxena et al., 2020).

The ACMS effectively prevents fraud. Clients generate a chaincode upon initiation of a transaction. The signature is verified by endorsing peers, and the endorsements are collected and sent to the ordering services, where transaction validation is the last step (Kumar et al., 2019).

### Product distribution

The presence of multiple dealers and intermediaries presents an opportunity for malpractice that undermines supply chain efficiency. Blockchain has been hailed for preventing the circulation of poor-quality pharmaceuticals (Hulea et al., 2018). Substandard pharmaceuticals are sequestered, and their entry into the pharmaceutical supply chain is investigated. Ledger systems, chaincodes, and serialization*,* in which serial numbers are assigned to pharmaceutical products to enable identification and differentiation, are used to facilitate pharmaceutical distribution*.* Blockchain information is tightly regulated to avoid unauthorized access that could jeopardize security systems (Dwivedi, Amin & Vollala, 2020). The Internet of Things (IoT) in the pharmaceutical distribution system enhances efficiency (Botcha, Chakravarthy & Anurag, 2019).

### Tracking and tracing

Naturally, goods in transit should be tracked and traced from dispatch to destination. Delivery delays hamper business operations in general, but problems can lead to loss of life or exacerbation of illness in the pharmaceutical and health industries. Blockchain technology has been applied to the pharmaceutical supply chain (Schöner et al., 2020). Drug tracking and traceability are essential for business operations, patient health, and regulatory compliance. With elaborate and secure tracking and tracing technologies, goods in transit are delivered on time to the desired destinations, enabling uninterrupted pharmaceutical business and patient management. A secured international registry was created to facilitate the global distribution of drugs. Although the technology provides enormous opportunities for large pharmaceutical firms, smaller firms should also benefit (Garankina et al., 2018).

### Safety and security

Since drugs are high-value commodities, security is necessary to protect them. The cryptographic features of blockchain technology are the basis of safety and security in the pharmaceutical industry (Sinclair, Shahriar & Zhang, 2019). Tracing and tracking capabilities satisfy regulatory requirements, and cryptographic technology enhances drug security (Plotnikov & Kuznetsova, 2018). Security is bolstered against theft and the introduction of counterfeit pharmaceuticals. Unauthorized drug modifications are also mitigated, blocking opportunistic pharmaceutical stakeholders who modify drugs and, in this way, undermine quality. Supply chain systems are governed based on stringent safety and security measures that raise an alarm when breached (Plotnikov & Kuznetsova, 2018).

## Review Methodology

### Research question

We used a previously published methodology to define our research question (Liberati et al., 2009). In this section, we elaborate on our selection of the research questions listed in the introduction.

RQ1 aims to review previous studies of blockchain technology in the pharmaceutical industry. We focused on studies that have developed proof-of-concept blockchain frameworks in the pharmaceutical industry. Many white papers and vision papers have been published on the use of blockchain technology in the pharmaceutical industry, but our primary goal was to present insights on real-world applications.

RQ2 defined four primary areas in which blockchain technology has been applied in the pharmaceutical industry: counterfeit prevention, product distribution, tracking and tracing, and safety and security. In addition, RQ3 aims to identify the limitations associated with pharmaceutical industry-based blockchain research. Finally, RQ4 explores future research directions proposed in the literature.

### Study protocol and workflow

**Figure 4:**
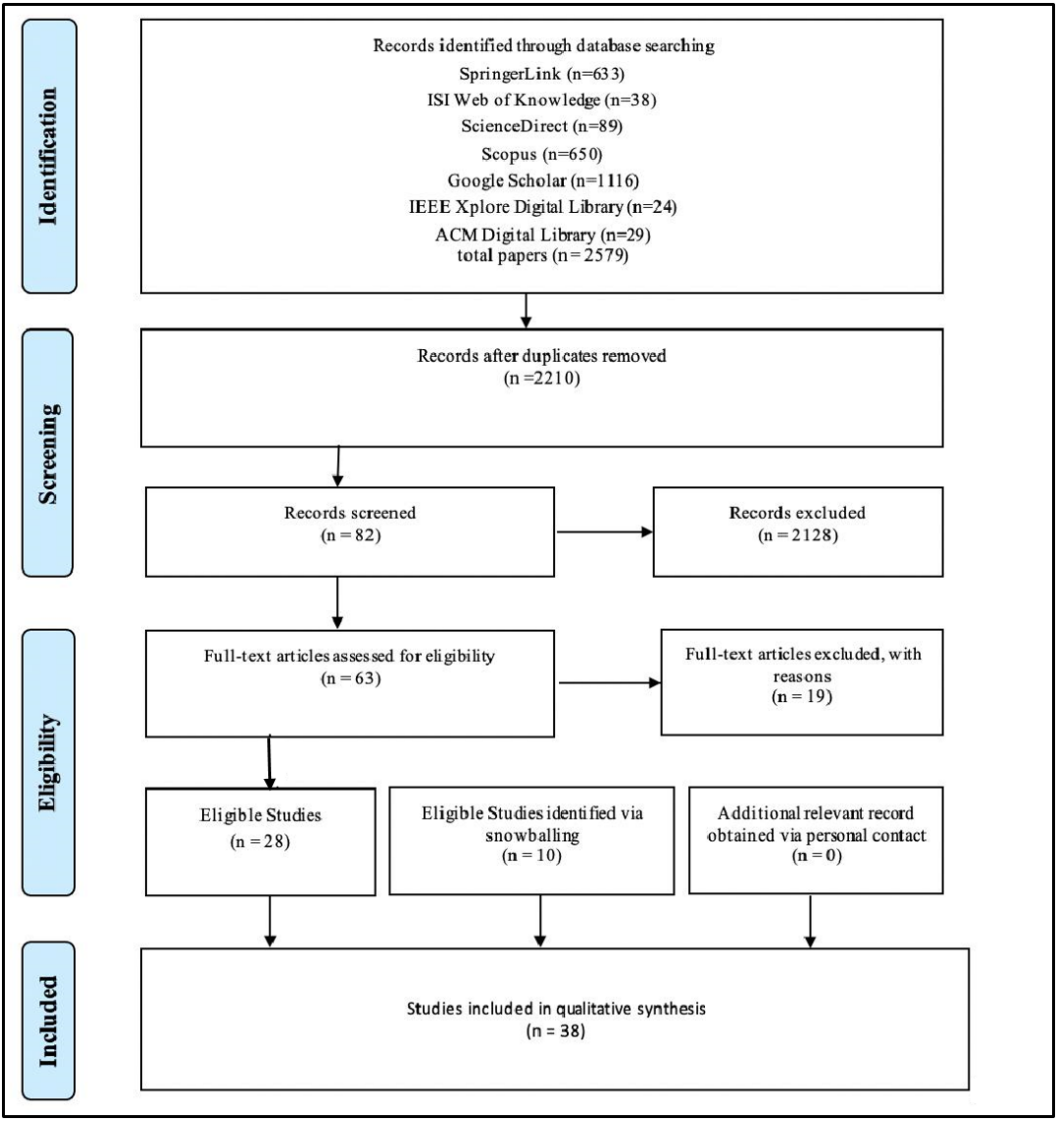
Preferred reporting items for systemic literature review and meta-analyses (PRISMA) statement adopted from Liberati et al. (2009).

The proposed review is based on the Preferred Reporting Items for Systematic Reviews and Meta-Analyses (PRISMA) statement (Liberati et al., 2009). The process is shown in Fig. 4.

### Inclusion and exclusion criteria

To be eligible for inclusion in this SLR, articles needed to meet the seven criteria shown in Table 1.

**Table 1:** Inclusion and exclusion criteria.

Inclusion	Exclusion
The paper should be written in English	Papers written in other languages
The paper should be a research article	Report, poster, presentations, etc.
The timeframe is (2016–2021)	Any article published before 2016
Papers related to computer science and related fields	Medical papers or other fields

### Information sources and search process

Seven databases were searched to identify relevant articles: namely, SpringerLink, Web of Science, ScienceDirect, Scopus, Google Scholar, IEEE Xplore Digital Library, and ACM Digital Library. In addition, the search keywords were:

• Blockchain
• Smart Contract
• Smart Contracts
• Distributed Ledger Technology (DLT)
• Pharma
• Drug
• Medicine
• Prescription

The final search term used for all seven databases was (Blockchain OR DLT OR “Smart Contract” OR “Smart Contracts” OR “Distributed Ledger Technology”) AND (Pharma* OR Drug* OR Medicine* OR Prescription*).

### Study selection and data collection

Seven researchers performed the data collection process, and the rest of the team provided guidance, support, and process verification. After extracting studies from the databases, the researchers independently screened each study’s title and abstract for eligibility. Eligibility disputes were settled by discussion until consensus was reached. The complete texts of the identified potentially eligible studies were retrieved and independently assessed for suitability.

First, we searched for relevant studies addressing blockchain technology in the pharmaceutical industry by entering the search term (see ‘Tracking and Tracing’) into each database’s search engine. In turn, the results were exported to a reference management system (in this case, Mendeley: (https://www.mendeley.com/) to eliminate duplicate entries. The papers were exported from Mendeley into Rayyan (Ouzzani et al., 2016), a tool that facilitates collaboration on screening and selecting research papers by multiple users. It is a free web and mobile app that uses semi-automated methods to expedite the initial screening of abstracts and titles (Ouzzani et al., 2016). Seven researchers independently screened the papers in Rayyan and scored them as “Include,” “Exclude,” or “Maybe.” The team met to discuss and reach a consensus on the 27 papers marked as “Maybe,” identifying 10 papers for inclusion and excluding the remaining 17. The final count at the end of this step was 82 papers marked for inclusion and 2,128 excluded.

Quality assessment was applied to the remaining 82 papers by using a modified method described elsewhere (Table 2) (Liao et al., 2020). The quality assessment yielded 28 papers for inclusion in the final analysis. A ranking system was applied (Erokhin, Koshechkin & Ryabkov, 2020) in which six questions were asked for each paper, where questions were scored as 1 for “yes,” 0.5 for “partially true,” and 0 for “no.” Papers with scores of 3 or more were retained.

**Table 2:** Quality assessment questions.

1. Is the aim of the research clearly stated?
2. Are the research methods clearly described?
3. Are the results of the research clearly stated?
4. Does the paper mention the impact of blockchain technology in the pharmaceutical industry?
5. Does the research propose an effective solution to prevent the circulation of counterfeit medicines?
6. Does the paper discuss study limitations?

After identifying the high-quality papers, we applied forward and backward snowballing (Jalali & Wohlin, 2012). This involved the following:

• Searching journal articles and conference papers to obtain a starting collection of papers
• Apply backward snowballing by checking the reference lists in step 1 and step 2 for related studies (iterate until no new papers are identified)
• Apply forward snowballing by finding papers that reference the studies

The articles retrieved from forward and backward snowballing (*n* = 10) were also tested against the quality assessment questions. This yielded a final count of 38 relevant articles for the SLR.

## Results

We independently classified the 38 selected studies by topic and research focus to respond to RQ1 and RQ2. After reading the studies, the team met for several rounds of discussion to finalize their classification(s). Table 3 shows the most frequently addressed topics in five categories: (1) counterfeit drug prevention, (2) product distribution, (3) tracking and tracing, (4) safety and security, and (5) other.

**Table 3:** Classification.

Topic	Description	Papers
Counterfeit drug prevention	Products that are intentionally produced and faked the label of their identity to make it appear like real products.	1, 7, 10, 11, 12, 13, 14, 18, 19, 20, 21, 22, 23, 24, 25, 26, 27
Product distribution	Distributing the drugs to all participants with a supply chain, starting from the pharmaceutical industry to wholesalers, hospitals, or pharmacies.	1, 5, 6, 7, 8, 9, 17
Tracking & tracing	Tracking all phases of drug production from manufacturer to consumer and quality assurance.	1, 6, 11, 12, 16, 19, 20, 22, 24, 26, 27, 30, 32, 33, 34, 35
Safety and security	Protecting data from modification, deletion, and manipulation, and transmitting it safely and reliably throughout the network.	1, 10, 25, 37, 38, 39, 40, 41
Others	Topics not covered by the category definitions, such as data governance, data quality, pharmaceutical turnover, and prescription drug monitoring.	8, 9, 14, 22, 34, 42, 43, 44, 45, 46
Others/Data governance	Principles and practices that guarantee high quality throughout the entire data lifecycle.	8, 22, 34, 42
Others/Data quality	An evaluation of the state of data based on accuracy, completeness, consistency, reliability, and whether it is up to date.	8, 9, 14, 30, 43, 44
Others/pharmaceutical turnover	The volume of a metabolized or refined substance, usually within a specified period.	45
Others/Prescription drug monitoring	Monitoring and documenting prescriptions of controlled substances.	46

**Figure 5:**
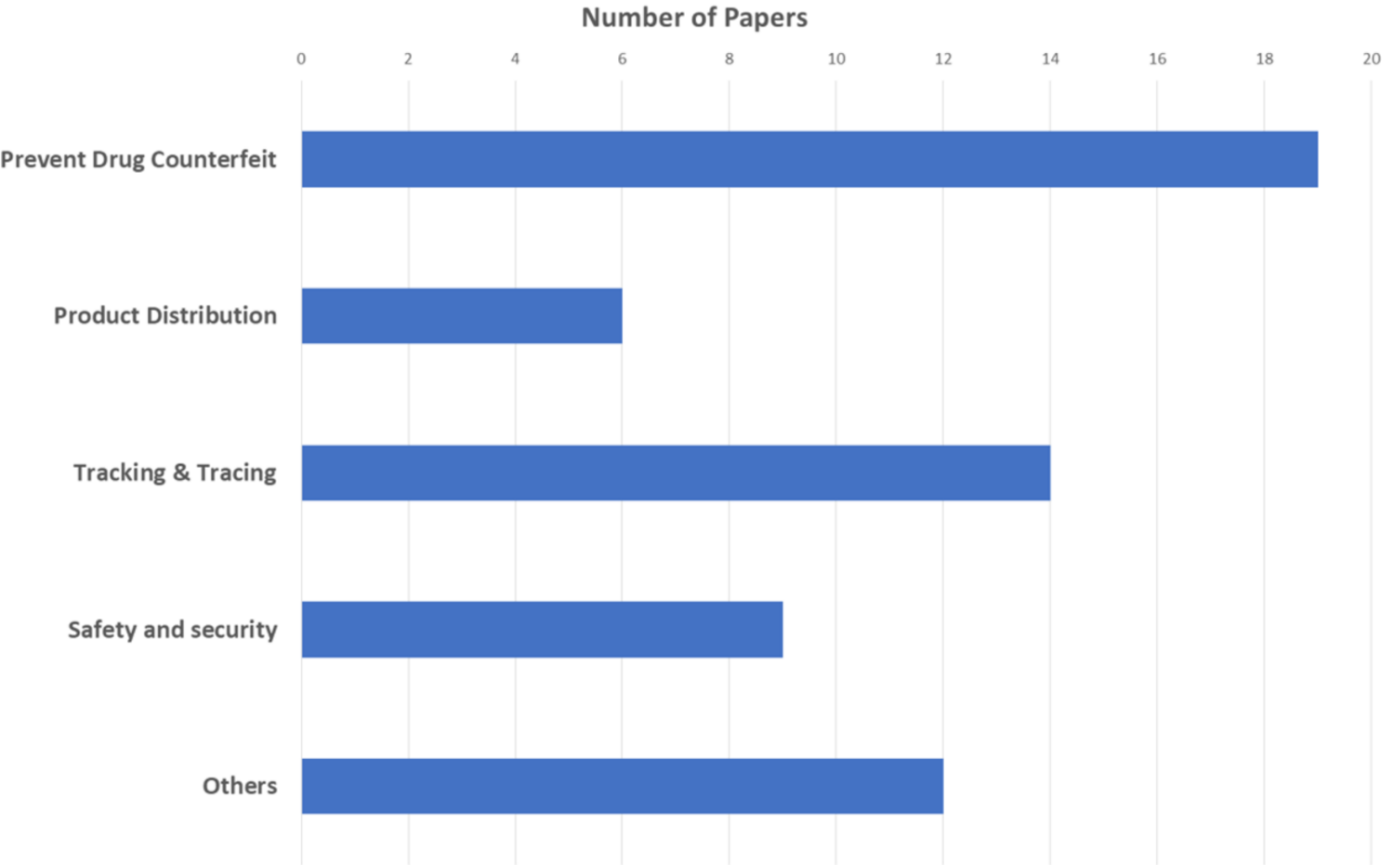
Summary of the main categories and their techniques.

The most frequent category was counterfeit drug prevention (17/38, 45%), which is consistent with the fact that counterfeit drug prevention is the primary objective of the pharmaceutical industry (Fig. 5). Newer topics in the field included data governance, data quality, pharmaceutical turnover, and prescription drug monitoring.

## Discussion

**Figure 6:**
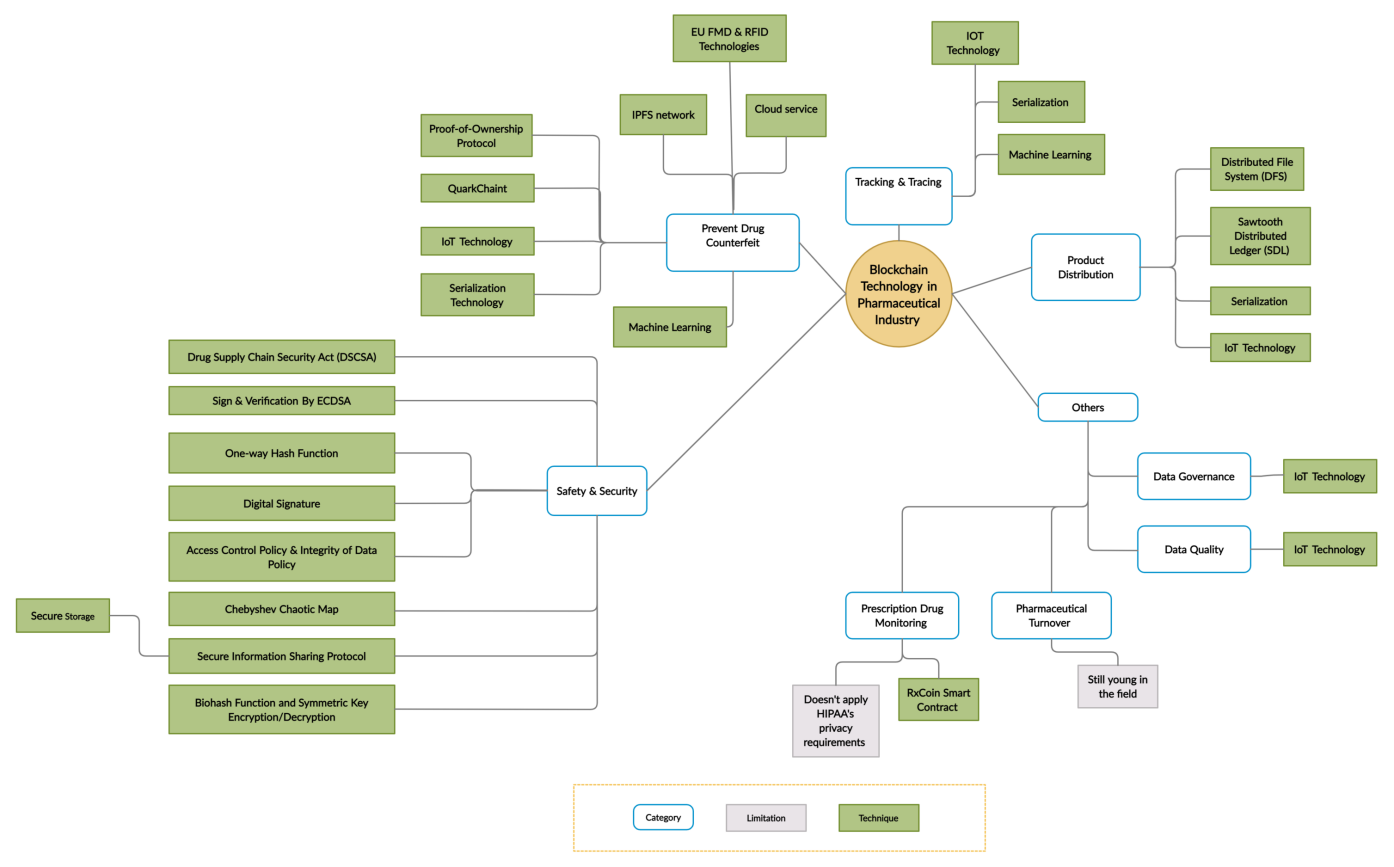
Papers classified by topic.

Multiple studies have investigated the use of blockchain technology in the pharmaceutical industry. However, no SLR has been conducted that discusses the issues, challenges, and solutions relating to the adoption of blockchain technology in the pharmaceutical industry. Therefore, our goal is to provide real-world insight into the implementation of blockchain technology in the pharmaceutical industry. We also defined the pharmaceutical industry’s primary areas, wherein blockchain has been applied in counterfeit drug prevention, product distribution, tracking and tracing, and safety and security (Fig. 6).

### Classification of papers

#### Counterfeit drug prevention

Patients, as well as the pharmaceutical industry as a whole, suffer from the trade of counterfeit drugs, particularly given that these drugs may be ineffective or toxic. The problem of counterfeit drugs is most severe in developing countries. According to the Health Research Funding Organization, nearly 10–30% of the drugs in developing countries are fake. Additionally, the World Health Organization stated that around 30% of all medicines sold in Africa, Asia, and Latin America are counterfeit (Jamil et al., 2019). In this section, a discussion is presented of blockchain-based solutions that have been proposed in the literature for counterfeit drug prevention.

#### Percentage of counterfeit drugs 8

Abbas et al. (2020) analyzed the vulnerability of the traditional drug supply chain, which is difficult to track and creates opportunities for counterfeiters to introduce fraudulent products into the market. A drug supply chain management and recommendation system based on blockchain and machine learning were proposed. The system incorporates blockchain-based drug supply chain management and a machine learning-based drug recommendation system integrated through REST APIs.

In the research undertaken by Jamil et al. (2019), a system for drug supply chain management based on a blockchain technology deployed in Hyperledger Fabric was proposed, relying on smart contracts to support supply chain safety and security.

Chiacchio et al. (2020) proposed a decentralized application based on blockchain and serialization technologies to support immutable traceability. In addition, Xie et al. (2019) proposed a simulated blockchain-based framework that supports the biopharmaceutical supply chain, including the prevention of counterfeits. The proposed framework is based on proof of authority and smart contracts, and a preliminary empirical study was undertaken to evaluate it.

Another group of authors proposed a roadmap for how blockchain technology can help participating stakeholders prevent counterfeit drugs from entering the supply chain (Soundarya, Pandey & Dhanalakshmi 2018). Also, a practical anti-counterfeit medicine management system based on blockchain technology was proposed by Pham, Tran & Nakashima (2019) with the aim of resisting the cloning of drugs and improving practical applicability. The proposed solution is based on the Ethereum blockchain and IPFS networks to support tamper-proof traceability. Raj, Rai & Agarwal (2019) proposed a solution to prevent counterfeit drugs from entering the pharmaceutical supply chain. Blockchains were used to establish proof of ownership, which is important because, before reaching patients, drug ownership changes from the manufacturer to the distributor to the pharmacist. The authors described the challenge of easily cloning RFID tags, highlighted blockchain’s ability to overcome this challenge, and added more features to the chain. A permissioned blockchain was designed to ensure that only trusted parties could join the network.

Repeated changes in the ownership of a drug give rise to vulnerabilities because counterfeiters can exploit these changes to inject counterfeit drugs into the market. Therefore, one prior study developed a form of quantitative analysis to propose the potential for blockchain-based control of India’s supply chain (Kumar et al., 2019). PharmaCrypt, a blockchain-based tool to prevent counterfeiting, was also developed in another study based on feedback from interviews with pharmaceutical and blockchain industry experts, which helped to define the software requirements (Saxena et al., 2020). Other researchers have proposed an IoT-based blockchain implementation to achieve drug governance for the pharmaceutical supply chain, the idea being to maintain an immutable record of transactions and prevent fraud (Ahmadi et al., 2020).

The Gcoin blockchain was developed to prevent the double-spending problem for drugs (*i.e.,* “spending” the same drug twice), which is similar to digital currencies (Tseng et al., 2018). Gcoin prevents a drug from being replaced with a counterfeit after blockchain registry, where the drug-specific QR code scanning technology is used to verify authenticity. A QR code is generated at the moment each drug is produced (Adsul et al., 2020).

Another paper proposed a counterfeit prevention system that involved tracking drugs from the manufacturer until they reach the end-user (Makarov & Pisarenko, 2019). In this model, drug manufacturers control and record all transactions on the blockchain. Any attempted fraud is revealed by a comparison of the details of data previously stored on the blockchain with what is entered, where mismatches reveal fake medications. The proof of work (PoW) consensus algorithm was used by the authors as the drug manufacturers are the data miners.

Elsewhere in the literature, a government-controlled medicine smart contract was developed to prevent counterfeiting by classifying retail medications with a serial number, while non-retail medications were classified with a QR code (Adsul et al., 2020; Tseng et al., 2018). The classification was achieved by implementing the GS1-128 standard for managing product ownership. Another proposed system relies on QR code scanning as the final verification of drug authenticity after the drug manufacturer has been registered on the blockchain through the Food and Drug Authority (Ying et al., 2019). This proposal implies that only authorized companies can register QR codes.

An IoT solution involves placing a wireless sensor inside drug packaging to enable real-time location tracking to the point of destination (Plotnikov & Kuznetsova, 2018). A similar IoT solution involves placing a chip-enabled label on the drug package. Another tracking proposal involves registering a unique identifier for each drug, where all stakeholders can use the identifier to query the handling and history of the drug (Zhu et al., 2020).

### Product distribution

In supply chain management, products are transferred between multiple parties, none of whom are aware of any other exchange that occurred within the chain. Significantly, the drug supply chain lacks the infrastructure to ensure comprehensive manufacturer-to-end-user tracking (Sahoo et al., 2019).

The manufacturer first distributes drugs to wholesalers, who then distribute the drugs to hospitals or pharmacies. Every transaction in this process should be tracked. Blockchain is a suitable technology to solve distribution process problems such as the circulation of counterfeit or illegal drugs (Fernando et al., 2020). Serialization may be used to allow reconstruction of the packaging hierarchy and product history (Dwivedi, Amin & Vollala, 2020).

One prototype identified four nodes: the manufacturer, the wholesaler, the retailer, and the Food and Drug Administration (FDA) (Sylim et al., 0000). The FDA is responsible for authorizing manufacturers and storing the authorization as a smart contract on a blockchain network. The system’s back-end is a distributed file system that supports a private blockchain network. On the front-end, it is stored in each node, and the interfaces reflect only the part of the supply chain in which a given user account is involved. There is a section for displaying performed transactions and the distribution chains, as well as a dashboard for anomaly detection. Each participant will create drug manufacture pedigrees, shipping pedigrees, and receipt pedigrees, sign them, and append them to the pedigree that is visible along the supply chain.

Other supply chain applications have focused on logistics to ensure quality, which is the practical management of products from the central unit to delivery. The proposed modum.io monitors transport data by using IoT sensors with blockchain technology (Bocek et al., 2017). A smartphone or tablet is preferred because it is the most convenient way to capture and scan barcodes. Another application is on pharmaceutical cold chain management, where a framework was proposed in Hulea et al. (2018) to track products during the distribution process, along with the supply chain, to ensure the delivery of information to all stakeholders. The Sawtooth Distributed Ledger is a permissioned blockchain where only approved participants can validate transactions or any change in network state.

### Tracking and tracing

The distribution of substandard and counterfeit drugs has led to deaths, prompting government agencies worldwide to implement trace and track systems to oversee pharmacy supply chains (Sylim et al., 0000). Blockchain provides new opportunities to ensure the traceability of medications (Raj, Rai & Agarwal, 2019). Blockchain also enables quality assurance and tracking through all drug production phases from manufacturer to consumer (Jangir et al., 2019; Ahmadi et al., 2020; Sahoo et al., 2019; Surjandy et al., 2019; Sahoo, Singhar & Sahoo, 2020).

Different approaches have been proposed to solve drug counterfeiting and improve pharmaceutical traceability (Raj, Rai & Agarwal, 2019), including the use of the Ethereum blockchain and distributed ledger technologies to manage the supply chain (Pham, Tran & Nakashima, 2019). This framework helps track drugs, user privacy, quality management, non-repudiation, transparency of the supplied drug, and demand-supply management with enhanced trust between users (Jangir et al., 2019). Drugledger, a scenario-oriented blockchain system for drug regulation and traceability, uses a peer-to-peer architecture (Huang, Wu & Long, 2018). System management for the drug supply chain is deployed using Hyperledger Fabric to continuously track drug delivery in the pharmaceutical industry (Abbas et al., 2020). A quantitative analysis was used in India to characterize key blockchain characteristics such as accountability, authenticity, and the ability to track drug information (Kumar et al., 2019). IoT devices have also been used to ensure data source authenticity and assess the current status of pharmaceutical products at any time and any place. Blockchain helps to mediate data storage and sharing, thereby guaranteeing that data are transparent and traceable (Shi, Yi & Kuang, 2019). Notably, the Italian pharmaceutical industry adopted a serialization regulation and blockchain-based technological solution (Dwivedi, Amin & Vollala, 2020).

### Safety and security

The design features of traditional drug supply chain management cannot transmit necessary information safely and reliably. In many cases, data can be deleted, modified, and tampered with easily. Many researchers have recently turned to blockchain technology as a way to transmit data through drug supply chains. The blockchain architecture consists of a set of immutable data blocks that are arranged in sequence and timestamped. Each block has a hash function, which is a digital fingerprint of data. Since all blockchain transactions are timestamped and immutable, blockchain technology can help preserve the integrity and reliability of drug data. In this section, we summarize how researchers have discussed various proposed blockchain-based solutions to keep drug data safe and reliable throughout the network.

Bera et al. (2020) examined how blockchain can be applied to meet the pharmaceutical supply chain’s security compliance requirements. They developed a prototype using blockchain to support the Drug Supply Chain Security Act requirements. The prototype was implemented using the Hyperledger Composer tool, modeling the various supply chain entities and access control rules. The result was faster development of blockchain applications and faster integration with supply chain businesses. Sahooet et al. (Xie et al., 2019) also discussed blockchain technology’s potential to prevent problems when managing the pharmaceutical supply chain transparently and securely; these authors extensively used the Elliptic Curve Digital Signature Algorithm (ECDSA). In their proposal, the drug supply chain relies on a trusted blockchain and IoT-guided network, where controlled details such as temperature and location can be recorded using wireless sensors and GPS devices attached to the packages. Pharmaceutical bottles are marked with serial numbers and the unique fingerprints of their manufacturers. Therefore, when a consumer finally purchases a drug, they can scan the label with a smartphone and learn everything about it.

Ying et al. (Bocek et al., 2017) proposed a system for supplying prescription drugs using blockchain technology. This architecture can protect patient privacy because a dynamic identity is provided, and an effective authentication protocol is designed between the participating parties. Furthermore, the architecture can meet the security requirements and computational requirements of healthcare systems, including authentication, secure data sharing, visibility, user privacy, and efficiency. Makarov and Pisarenko (Fernando, 2019) were also interested in data integrity; they proposed a blockchain model for drug production and supply. It traces the origin of raw materials and medicinal products to ensure the quality of any drugs entering the pharmacy warehouse. Under this system, the product manufacturer, pharmacy warehouse, and pharmacy have access to complete, reliable, and secure information about the origin and quality of the drugs registered on the blockchain. Jamil et al. (Huang, Wu & Long, 2018) contributed to preserving drug data by proposing a novel blockchain-based system for drug supply chain management. In their system, Hyperledger Fabric was used in a smart hospital to facilitate the secure handling of drug supply chain records.

Electronic prescriptions and patient information are efficiently stored and shared in a secure authorized network chain across various hospital departments. Smart contracts have been used to ensure the consistency of drug data and other health-related information, in addition to granting time-limited access to electronic drug records and electronic patient health records. An access control policy is also defined to authorize the request for transactions in the proposed system. The authors proposed a novel method for integrating and deploying a CouchDB for each node to avoid data duplication across the blockchain file system. Kim et al. (Hulea et al., 2018) used IPFS to store data to develop a patient-centered drug history recording system that could help collect drug history more conveniently and reliably using the blockchain, and also prevent tampering with drug data. The patients’ encrypted prescription data are stored in QR codes. The data hash value is stored in the blockchain to prevent data tampering and fraud. Dwivedi, Amin & Vollala (2020) proposed the blockchain-based PSCM to securely share information in a pharmaceutical supply chain system with smart contracts and consensus mechanisms. The proposed system also provides a mechanism to securely distribute the required cryptographic keys to all participants using smart contract technology. Every new transaction made in the network is stored in an immutable block and timestamped to trace the specific product in the chain end-to-end, thereby ensuring that the block details are not modified. Gürsoy et al. (Archa, Alangot & Achuthan, 2018) designed a proprietary smart contract to efficiently store and query pharmacogenomics data using the Ethereum blockchain with a multi-map, index-based approach. The nodes store each genomic note (a trio of genetic drugs with results) in a searchable mapping by a unique identifier, allowing for efficient storage and query time and space.

### Other

Other uses of blockchain in the pharmaceutical industry are described in this section.

a) *Data Governance*

Blockchain and IoT technology have been used to improve data governance and supply chains (Jalali & Wohlin, 2012). IoT has also been used to ensure medical product regulatory compliance (Ahmadi et al., 2020). The Drugledger system tracks traceability and regulations and has been used in vaccine production to ensure organizational and regulatory compliance (Sinclair, Shahriar & Zhang, 2019). In the future, the technology will also include vaccine circulation (Surjandy et al., 2019).

b) *Data Quality*

IoT technology has been used to ensure quality throughout the transport of medical products (such as temperature control) and to satisfy GDP regulations, where sensor devices control each parcel’s temperature during shipping (Ahmadi et al., 2020). Likewise, IoT can be applied to track the temperature of medical products during distribution, which informs all stakeholders to take action if needed (Tseng et al., 2018). Moreover, another IoT technology tracks drug authenticity (Sylim et al., 0000). Some studies have used it to detect low-quality drugs due to its power in data management (transparency and immutability) (Badhotiya et al., 2021). Another system encourages different stakeholders to engage in transparent management throughout the recall process (Sahoo, Singhar & Sahoo, 2020). Recall management can improve efficiency and accountability, and it can also safeguard the credibility of product recall data. Most of the market does not have open management or tamper resistance, but stochastic simulations have been used to protect drug products from theft and temperature deviations (Liberati et al., 2009).

c) *Pharmaceutical Turnover*

Blockchain technology (in this case, Hyperledger Fabric) has been used to control pharmaceutical turnover, but its future utility in this function is so new as to be uncertain (Shi, Yi & Kuang, 2019).

d) *Prescription Drug Monitoring*

The RxCoin smart contract was adopted to combat the opioid crisis, which is based on a form of digital currency used to describe drugs electronically with Ethereum blockchain technology (Ying et al., 2019). RxCoin demonstrated the potential for prescribing drugs *via* the blockchain, and as a result, a database of prescription data was created. RxCoin’s components include data and function calls that provide the smart contract with the prescription function and mint. The main advantage of the RxCoin smart contract is how it handles authorized refills. RxCoin contracts do not show how the blockchain can be used to provide the functionality of a Prescription Drug Monitoring System (PDMP), nor do they consider HIPAA privacy requirements for personal health information stored on the blockchain. User interfaces for RxCoin are under development. In addition to using digital signatures as identities for members of the system, researchers are working diligently to harden RxCoin contracts to fill existing gaps.

## Research Challenges

Blockchain technology is a growing area of study. Although blockchain technology was once used exclusively in the financial field, it is now being applied to public services, IoT, reputation systems, and security services. The pharmaceutical industry is the latest field to leverage blockchain technology, where one of the most prominent application areas is the counterfeit problem. It is noteworthy that research activity focusing on the use of blockchains in the pharmaceutical industry has steadily increased over the past three years (Fig. 4). In this study, we identified multiple challenges that are currently facing the pharmaceutical industry. We discuss some of these challenges in this section, focusing in particular on those associated with blockchain applications, and we also present their solutions.

### Blockchain security

Some of the features that blockchain technology offers, primarily due to decentralization, are information security and privacy. However, this requires extra computational power. Around half of the power is spent in deceiving attacks by malicious users; otherwise, users can suffer losses from theft and the loss of goods due to a lack of robust tracking and tracing (Plotnikov & Kuznetsova, 2018). When blockchains are distributed, this creates a more complex environment that makes it difficult to overcome through fraudulent block transactions. Also, permissionless blockchains –in which any user can join –can also be secured using Hyperledger. Specifically, Hyperledger makes permissionless blockchains more secure and only allows the users who are involved in the transaction to join.

### Standardization

Blockchains can facilitate secure banking transactions globally, but a significant challenge relates to interoperability issues. To standardize blockchain operations, distributed ledger technology (DLT) can be utilized. The International Telecommunication Union (ITU) works to identify and standardize DLT applications and their services, but further research is needed to identify the best practices for implementation.

### Complexity of big data

**Figure 7:**
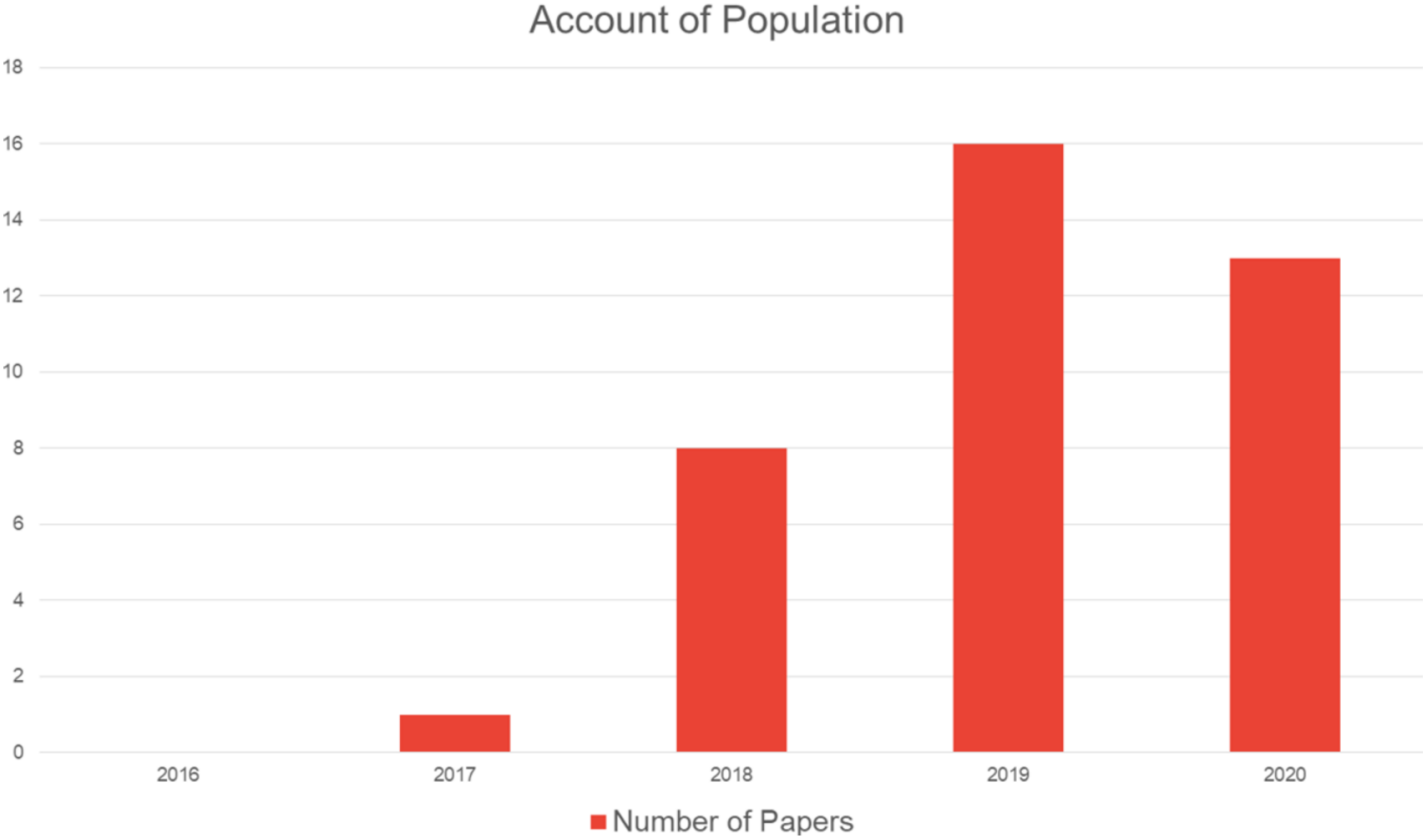
Paper distribution by year of publication.

Due to the use of cloud computing, edge computing, and smart IoT applications, the availability of data has grown substantially. Due to this enormous growth, there are many challenges associated with blockchain implementation, including data management, accessibility issues, data redundancy, and data privacy. They also suffer from malpractice and poorly functioning supply chains, and have sought to employ blockchains to harness operations and streamline tracing and tracking, medical transactions, and patient safety (Sinclair, Shahriar & Zhang, 2019). Therefore, the complexity of data must be handled in a blockchain by leveraging big data handling algorithms and other state-of-the-art techniques (Kim et al., 2019; Gürsoy, Brannon & Gerstein, 2020; Hu et al., 2019). Figure 7 shows the distribution of papers by the year of publication.

## Future research directions

Table 4 provides an overview of the limitations of the previous studies. Most data nowadays is big data, which has properties such as variability, volume, and complexity. Hence, data processing systems require greater computational capabilities. As we also know, decentralized distributed ledgers and blockchains require substantial computational power to provide better security, immutability, and increased transparency. For this reason, blockchain systems often suffer from computational complexity issues. Therefore, adaptive blockchain architectures should be designed that can provide better and faster services.

**Table 4:** Limitations of selected studies.

**Limitation & future research**	**Categories**	**Papers**
The mediator between patient and manufacturer has not been removed due to the absence of smart contracts in the proposed system.	Safety and security	1
The result of the proposed system may not reflect real-world performance.	Product distribution	7
Cannot track counterfeit drugs distributed through routes outside of official distribution chains.	Counterfeit drug prevention, Product distribution	7
Network size and performance were not tested in a real-world environment, and the accuracy of the machine learning modules was limited.	Counterfeit drug prevention	12
Network size limitations were not tested in a real-world deployment.	Counterfeit drug prevention	10
Noticeable latency because adding the QR code requires transaction confirmation, which is considered slow in Ethereum.	Counterfeit drug prevention	13
Challenges in adopting the blockchain technology for the pharmaceutical supply chain are discussed: 51% attacks, trust, transparency, scalability, integration, and cost.	Counterfeit drug prevention, Tracking & Tracing	18, 27
Immaturity of a still-emerging technology.	Counterfeit drug prevention	11
Total transactions per second limited to 1600; a lack of technical experts.	Counterfeit drug prevention, Tracking & Tracing	20
Scalability challenge.	Counterfeit drug prevention, Tracking and Tracing, Safety and Security	21, 27, 33, 39
Information authentication still needs verification.	Tracking and Tracing	21
Gas high-cost issue.	Tracking and Tracing, Safety and Security	13, 35
Large memory utilization.	Tracking and Tracing	21
Absence of universal guidelines, rules, and code to guide implementation of blockchain technology.	Tracking and Tracing	8
Many facets of the prototyping environment currently seem lacking.	Safety and Security	31

With the advent of 5G, an exponential increase has occurred in the number of edge and smart devices. This has made the implementation of blockchains more difficult, particularly given the diverse technological environment of 5G. Hence, there is a need for more decentralized, secure, private, transparent, interoperable, and immutable blockchain technology for the 5G landscape. Blockchain architectures must integrate with platforms and technologies such as software-defined networks, cloud computing, and mobile edge computing.

## Conclusions

Blockchain is an emerging technology that is expected to open up a promising future for various industries. This research focused on the case study of state-of-the-art blockchain technology in the pharmaceutical industry. A systematic literature review (SLR) was undertaken that involved 38 relevant papers.

The SLR’s results indicate that the included papers, taken together, covered the major topic areas of counterfeit drug prevention, drug distribution, tracking and tracing, safety and security, and other. Each category was explained and limitations were discussed to help future researchers identify gaps in this newly emerging field. In this SLR, we focused on proof of concept blockchain frameworks that have been developed in the pharmaceutical industry. We discussed many white papers and vision papers that have focused on the use of blockchain in the pharmaceutical industry, where the primary goal was to present insights on real-world applications. The SLR also identified the limitations associated with pharmaceutical industry-based blockchain research.

Another important aspect of this SLR is its discussion of the challenges facing blockchain applications in complex environments, such as those involving big data, edge computing, and software-defined networks. We also discussed future directions and the need for adaptive blockchain architectures that can cope with the computational complexity of big data and cloud computing.

## Additional Information and Declarations

### Data Availability

The following information was supplied regarding data availability: This study is a literature review.
